# Late Gadolinium Enhancement Cardiac Magnetic Resonance Imaging Post-robotic Radiosurgical Pulmonary Vein Isolation (RRPVI): First Case in the World

**DOI:** 10.7759/cureus.738

**Published:** 2016-08-15

**Authors:** Edgar Monroy, Jose Azpiri, Cuauhtémoc De La Peña, Carlos Cardona, Miguel Hinojosa, Rafael Zamarripa, Jose Assad

**Affiliations:** 1 Imaging, Hospital Christus Muguerza; 2 Cardiology, Hospital Christus Muguerza; 3 Oncology, Hospital Christus Muguerza

**Keywords:** atrial fibrillation, cardiac magnetic resonance, cardiac radiosurgery, pulmonary vein isolation, cyberheart, cyberknife

## Abstract

Pulmonary vein isolation using robotic radiosurgery system CyberKnife is a new non-invasive treatment of atrial fibrillation, currently in clinical phase. Robotic radiosurgical pulmonary vein isolation (RRPVI) uses stereotactic, non-invasive (painless) pinpoint radiation energy delivery to a small, precise area to accomplish ablation. The purpose of this report is to describe the finding of an increase in the enhancement of the left atrium demonstrated with the use of cardiac magnetic resonance imaging using late gadolinium enhancement (LGE-CMR) as a result of RRPVI in the first case in the world in humans using CyberKnife as a treatment for paroxysmal atrial fibrillation (PAF).

## Introduction

Atrial fibrillation (AF) is a disorder affecting 20 million people of all ages worldwide, with the incidence increasing with age. It is estimated that 15.9 million people in the USA alone will have the disease by 2050 [1]. AF is the leading cause of stroke, which is the third leading cause of death in the USA. The increased risk of stroke, heart failure, morbidity from anticoagulation therapy and complications of AF treatment increase the rate of hospitalizations. In the last decade, catheter ablation has provided an improvement in morbidity and quality of life and has significantly reduced long-term healthcare costs [2].

Despite recent progress in techniques, current catheter ablation strategies fall short of expectations [3-4]. In patients with paroxysmal and persistent AF, recurrence rates after one year following discontinuation of antiarrhythmic treatment are 70–80% and 50%, respectively. Furthermore, the incidence of significant complications with catheter ablation is approximately 6% [5].

During the past decade, cardiac magnetic resonance imaging using late gadolinium enhancement (LGE-CMR) has emerged as a well-established method for accurate and sensitive in vivo detection of left ventricular fibrosis in coronary artery disease and cardiomyopathies [6-7]. Higher spatial resolution LGE-CMR was subsequently extended to identifying scarring/fibrosis in the wall of the left atrium (LA) and pulmonary vein (PV) after pulmonary vein isolation (PVI) [8]. Studies suggest that quantification of post-PVI LA or PV scarring may play a role in determining  the risk of AF recurrence [9].

Catheter ablation is an endovascular approach. It consists of threading ablation catheters into the left atrium from the femoral vessels. It uses radiofrequency energy delivered by the catheter to accomplish point scarring and ablation. RRPVI, by contrast, uses stereotactic, non-invasive (painless) pinpoint radiation energy delivery to a small, precise area to accomplish ablation.

Robotic radiosurgical pulmonary vein isolation (RRPVI) is a new, non-invasive treatment of atrial fibrillation, currently in clinical phase. The purpose of this report is to describe the finding of an increase in the enhancement of the left atrium demonstrated with the use of LGE-CMR as a result of RRPVI in the first case in the world in humans using CyberKnife as a treatment for paroxysmal atrial fibrillation (PAF).

## Case presentation

A 59-year-old man presented with a history of dyspnea and palpitations secondary to PAF over a seven-year period. He had a history of ischemic stroke two years previous that neurologically resolved and was under treatment with anticoagulants and anti-arrhythmic drugs that generated side effects. Due to the adverse effects of the antiarrhythmic drugs and failure to alleviate the symptoms, his cardiologist proposed pulmonary vein ablation using CyberKnife due to the fact that the patient was hesitant about standard PVI catheter ablation, and the cardiologist was concerned and wanted to avoid any catheter manipulation within the left atrium that would be required with standard PVI catheter ablation.

The patient  was enrolled in a clinical trial in Mexico, under COFEPRIS, “CyberHeart treatment for refractory atrial fibrillation – a pilot study”. Funding was from the sponsor (CyberHeart, Inc.) and the hospital, Christus Muguerza, Monterrey, Mexico. The patient agreed to participate and was explained the nature and objectives of this study, and informed consent was formally obtained. No reference to the patient's identity was made at any stage during data analysis or in the report.

**Figure 1:**
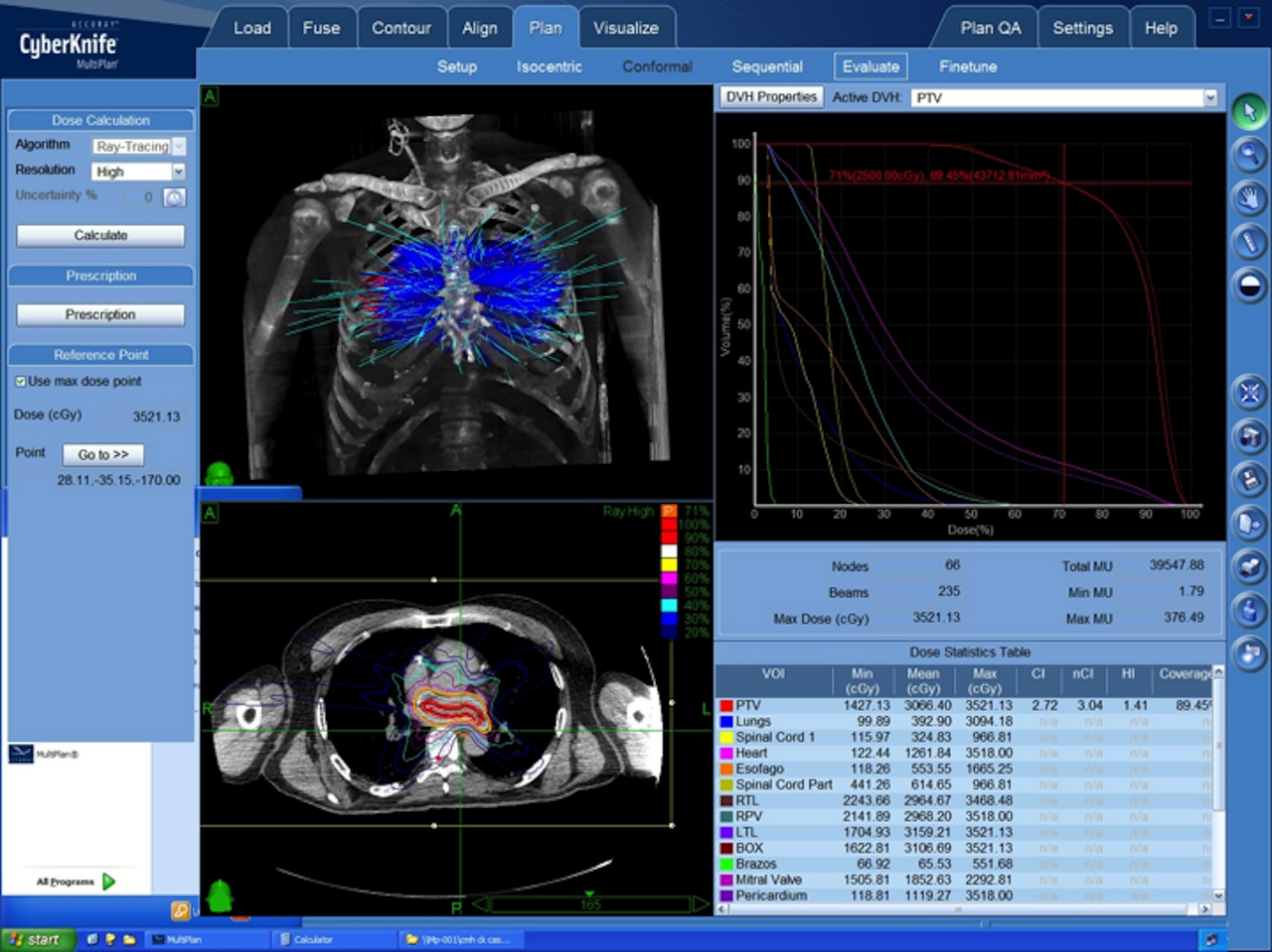
Multiplan Review Image Showing Beam Entries, Dose Volume Histogram, and Dose Statistics

The cardiologist contoured the desired ablation volumes in the atrial myocardium /pulmonary veins by isolating those structures in a 3D reconstruction of the CT data and drawing a ring around the pulmonary veins, which then produced a volume in the CT slices. This was done using computer-installed proprietary software (CyberHeart) and then exported to the Accuray MultiPlan® to continue with the planning process. A sequential optimization was used to deliver the dose to the target defined in the CyberHeart software while avoiding surrounding critical structures (Figure 1). Synchrony image guidance was used to compensate for respiratory motion during the treatment delivery.

The dose prescribed was 25 Gy to the 71% isodose line in one fraction (Table 1).

**Table 1:** Dose Statistics Table Including Contoured Volumes and Their Respective Mean and Max Doses VOI=Volume Of Interest. PTV=Planning Target Volume. RTL=Rigth Lung. LTL=Left Lung. RPV=Right Pulmonary Vein. CI=Comformality Index. nCI=new Conformality Index. HI=Homogeneity Index.

Dose Statistics Table							
Delivery System:		R235 1100-154811237190860800					
Collimator:		Iris					
Patient Name:		RRGG					
Medical ID:		141036					
Plan Name:		IRIS_FNL					
VOI	Min (cGy)	Mean (cGy)	Max (cGy)	CI	nCI	HI	Coverage (%)
PTV	1427.13	3,066.40	3521.13	2.72	3.04	1.41	89.45
Lungs	99.89	392.90	3094.18	n/a	n/a	n/a	n/a
Spinal Cord 1	115.97	324.83	966.81	n/a	n/a	n/a	n/a
Heart	122.44	1,261.84	3518.00	n/a	n/a	n/a	n/a
Esophagus	118.26	553.55	1665.25	n/a	n/a	n/a	n/a
Spinal Cord Partial	441.26	614.65	966.81	n/a	n/a	n/a	n/a
RTL	2243.66	2,964.67	3468.48	n/a	n/a	n/a	n/a
RPV	2141.89	2,968.20	3518.00	n/a	n/a	n/a	n/a
LTL	1704.93	3,159.21	3521.13	n/a	n/a	n/a	n/a
BOX	1622.81	3,106.69	3521.13	n/a	n/a	n/a	n/a
Skin	66.64	240.72	3521.13	n/a	n/a	n/a	n/a
Arms	66.92	89.01	551.68	n/a	n/a	n/a	n/a
Mitral Valve	1505.81	1,852.63	2292.81	n/a	n/a	n/a	n/a
Pericardium	118.81	1,119.27	3518.00	n/a	n/a	n/a	n/a
Ventricles	122.89	780.45	2604.30	n/a	n/a	n/a	n/a
Coronary arteries	1061.59	1,841.94	2766.41	n/a	n/a	n/a	n/a
Trachea	117.16	396.69	2411.61	n/a	n/a	n/a	n/a

The day after the cyberheart intervention, the patient developed paroxysmal atrial fibrillation that reverted to sinus rhythm with amiodarone IV infusion. He then stayed in sinus rhythm and was evaluated clinically every four to eight weeks until six months after the procedure when he developed permanent atrial fibrillation. He was cardioverted electrically once but returned to atrial fibrillation; therefore, we elected to control the rate with beta blocker and anticoagulate him with rivaroxaban. He has been asymptomatic since, though in atrial fibrillation. There were no esophageal, respiratory, or pericardial symptoms at the last clinic visit one month prior to this writing. LGE-CMR on a 1.5 T imaging system (GE Healthcare, Optima MR450w) was performed before and one year after RRPVI.

### MRI technique used

CMR was performed one day before and one year after PVI on a 1.5 T imaging system (GE Healthcare, Optima MR450w) with a dedicated 32-channel cardiovascular coil placed over the chest of the patient in the supine position. ECG monitoring was performed during the entire examination via an integrated MR-compatible monitoring system. After acquisition of scout views, dynamic axial, short- and long-axis images of the left atrium were acquired using a segmented k-space steady-state free-precession pulse sequence (repetition time: 4.2 ms, echo time: 1.8 ms, in-plane spatial resolution: 1.4 x 1.4 mm, slice thickness: 6 mm). Gadopentetate dimeglumine (Magnevist®, Bayer Schering Pharma AG, Berlin, Germany) was administered at 0.2 mmol per kilogram of body weight via an antecubital venous access. The administration of contrast was followed by a 20 ml saline injection. Delayed enhancement imaging was started ten minutes after injection of the contrast. No parallel imaging technique was used. Optimization of inversion time was performed using a cine inversion recovery (Cine-IR) done in a single breath-hold. This pulse sequence is used to determine the correct inversion time (TI) to null the signal intensity of normal myocardium. Sequence parameters of the Cine-IR sequence include field of view (FOV): 400 × 320 mm, matrix: 128 × 128, slice thickness: 6 mm, repetition time (TR): 4.5 ms, and echo time (TE): 2 ms.

All delayed enhancement imaging was performed using prospective ECG-triggering in the breath-hold technique. Data acquisition was performed during mid-diastole, which was estimated by a time window of minimal cardiac motion. Ten minutes after the injection of the contrast, a standard two-dimensional delayed enhancement imaging was performed using an inversion-recovery sequence on axial short- and long-axis images of the LA at matching cine-image slice locations with the following parameters: TR/TE: 4.8/1.5 ms, in-plane spatial resolution: 1.4-2.2 mm, bandwidth: 131 Hz per pixel, flip angle: 25°, a typical FOV of 390 × 310 mm, matrix: 224 × 128, number of signal averages: 2, slice thickness: 6 mm, inversion time: 237 ms, and breath-hold time: 9 seconds.

### Data analysis

The image analysis was performed using Volume Share 2 software (GE Healthcare Advantage Workstation 4.4) and EchoPixel visualization tool (EchoPixel Inc., Mountain View, CA).

The images were assessed by two independent observers who conducted a visual qualitative and descriptive analysis of areas with increased signal in the circumferential extent of the LA not observed in cardiac magnetic resonance prior to the treatment with CyberKnife. These areas were considered as a possible scar as a result of PVI in the images of LGE-CMR. Discrepancies were resolved by consensus.

### Findings on images

**Figure 2:**
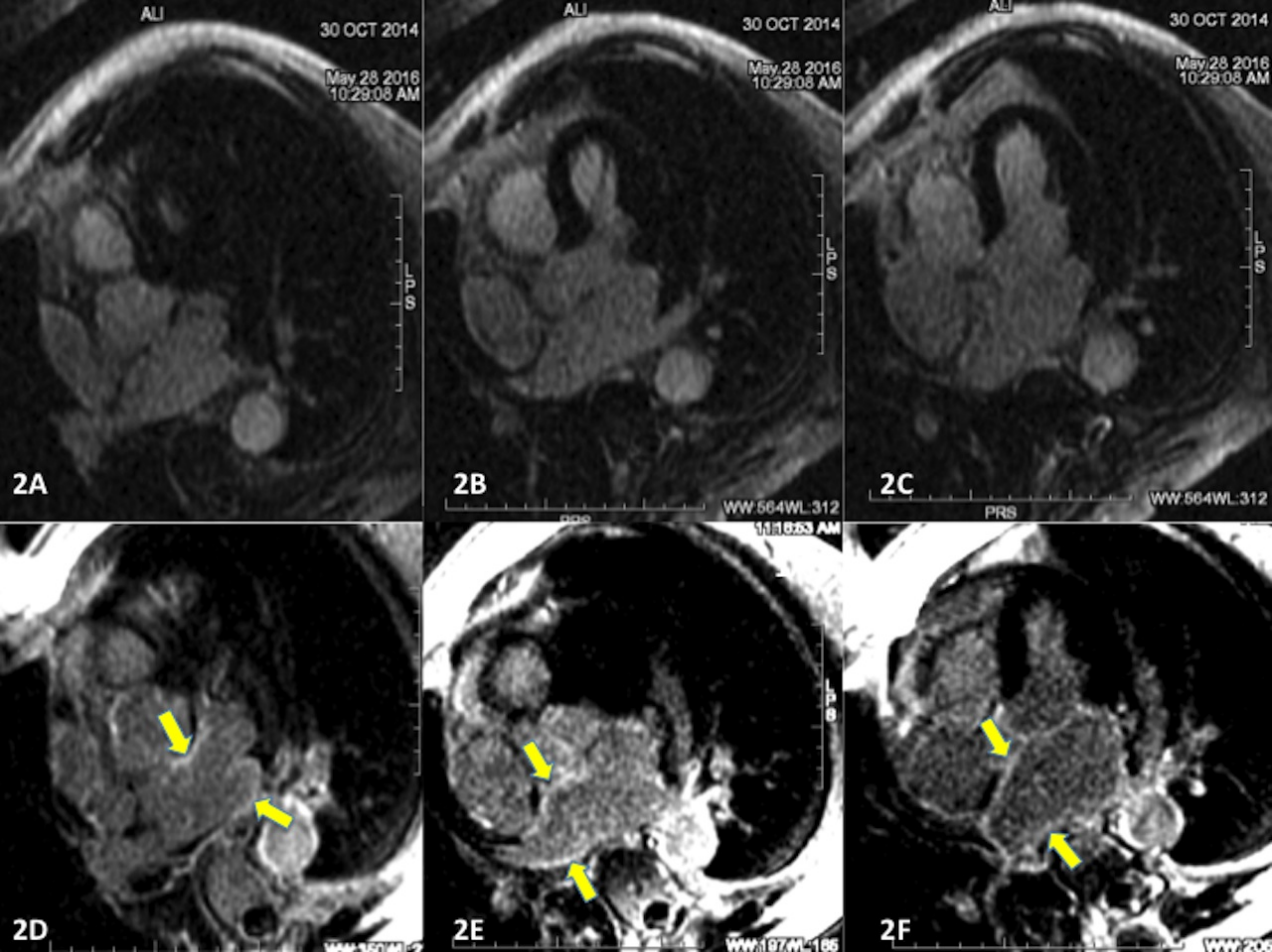
Delayed Enhancement Inversion-Recovery Images Before and After Treatment with CyberKnife 2A, 2B, and 2C: Axial 2-dimensional delayed enhancement inversion-recovery images pre-isolation of pulmonary veins. 2D, 2E, and 2F: Axial 2-dimensional delayed enhancement inversion-recovery images acquired one year after isolation of pulmary veins. Delayed enhancement is not observed before ablation but is readily observed after ablation (arrows in 2D, 2E, and 2F).

Axial short- and long-axis images of the LA were analyzed comparing images obtained one day before and one year after PVI. New sites of delayed enhancement were identified using visual qualitative analysis as areas with increased signal in the circumferential extent of the LA around pulmonary veins, base of left atrial appendage, roof of the left atrium and near the coronary sinus, not observed in cardiac magnetic resonance prior to the treatment with CyberKnife (Figure 2).

**Figure 3:**
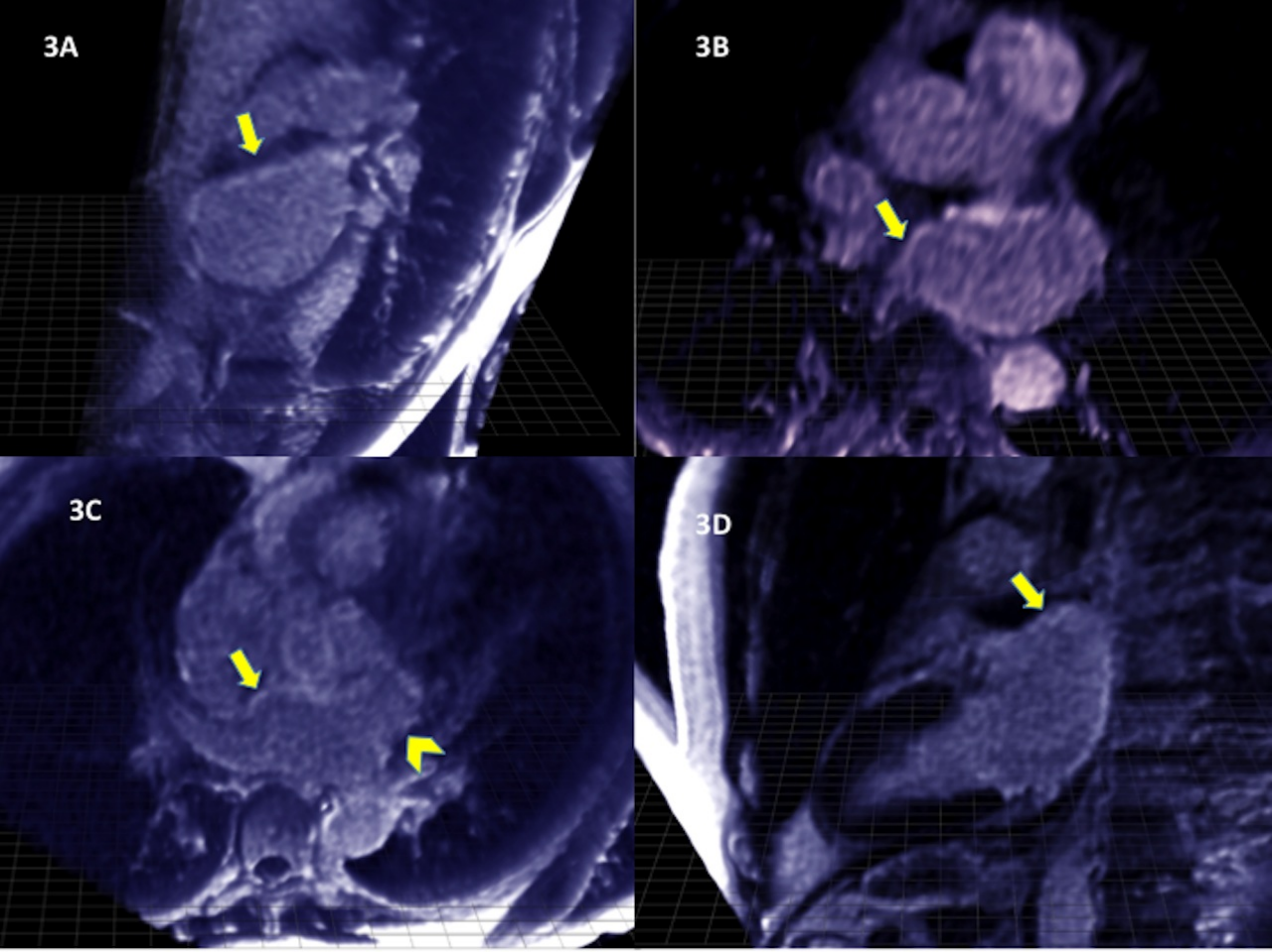
Echopixel Delayed Enhancement Inversion-Recovery Images After Treatment with CyberKnife 3A, 3B, 3C and 3D: delayed enhancement inversion-recovery images one year after isolation of pulmary veins using Echopixel to highlight the observed findings consistent with fibrosis around pulmonary veins (arrows in 3B and 3C), base of left atrial appendage (arrowhead in 3C), roof of left atrium (arrows in 3A and 3D), and near coronary sinus, subsequent to treatment with CyberKnife.

EchoPixel visualization tool was used to highlight the observed findings consistent with new sites of delayed enhancement around pulmonary veins, base of left atrial appendage, roof of the left atrium, and near the coronary sinus, subsequent to the treatment with CyberKnife (Figure 3).

## Discussion

Different groups of researchers indeed have examined multiple imaging modalities to personalize the strategy of atrial fibrillation ablation, to understand which atrial substrate is likely to improve post ablation, and to assess the efficacy of different ablation approaches and methods.

Karim et al., attempted to standardize and quantify scars from delayed enhanced MR images. The intent was to visualize scars on the left atrium in order to identify and demonstrate gaps in ablation patterns that could be the source of failed prior arrhythmia ablation attempts. Clinical datasets were examined to quantify scars such that an accurate predictor of a successful radiofrequency catheter ablation could be accomplished [10].

Subsequently in his review article, Gu et al., reviewed the status of MR imaging and catheter ablation for atrial fibrillation. It is noted that in prior studies, patients with extensive scarring at three months post ablation indeed had lower atrial fibrillation occurrence rates. Unfortunately, further work with MRI still has not yet identified ‘gaps’ in ablation line creation by radiofrequency. The goal would be to identify sites for repeat procedures, and therefore to achieve ablation success [11].

We consider the findings in the images of LGE-CMR in this case are evidence of paramount importance to demonstrate the concept of isolation of the pulmonary veins using the CyberKnife/CyberHeart. However, the patient came back in atrial fibrillation and has been on medical therapy for the last year. The findings in the LGE-CMR did show increase in the enhancement of the left atrium (that may correspond to the development of a scar) as a result of the CyberKnife treatment; however, we cannot yet prove these findings have indeed modified in the midterm follow-up with the recurrence/prognosis of atrial fibrillation in this patient.

CyberKnife/CyberHeart have undertaken a new approach to atrial arrhythmia ablation using radiosurgery.  In this first in man study, we wished to ascertain if delayed enhanced MRI would be useful to assess and quantify any left atrial scars, and to learn if scars could be correlated with the planned sites for non-invasive ablation. Future studies will compare pre- and post-ablation scans to learn if scars observed correlate with clinical outcomes.

## Conclusions

Cardiac radiosurgery potentially offers a promising, minimally non-invasive treatment option to patients with paroxysmal atrial fibrillation. Further studies are needed to document that the use of LGE-CMR is a key element to support the use of RRPVI in paroxysmal atrial fibrillation and demonstrate the effectiveness of CyberKnife therapy.

Future patients will undergo both pre- and post-CMR imaging using 3D acquisition method and software quantification.
